# Dynein and MEL-28 contribute in parallel to oogenic maturity in *C. elegans*

**DOI:** 10.17912/micropub.biology.000421

**Published:** 2021-07-27

**Authors:** Jay Gandhi, Giulia Crosio, Anita G. Fernandez

**Affiliations:** 1 Department of Biology, Fairfield University, 1073 N. Benson Rd., Fairfield, CT USA

## Abstract

*dhc-1(or283ts); mel-28(t1684)* double mutants have a severely reduced brood size compared to the wild-type and compared to each single mutant. To determine if this low-fecundity phenotype is associated with oocyte maturity defects, we used markers to assess the maturity of oocytes in the proximal gonad. We studied phosphorylated histone H3, a marker normally associated with mature oocytes, and DAO-5, a nucleolar marker normally associated with immature oocytes. We found that in the double mutants, the oocyte occupying the -1 position frequently retains DAO-5 and fails to accumulate phosphorylated histone H3. This suggests that the simultaneous disruption of dynein and MEL-28 can lead to failure of the oocyte maturity program.

**Figure 1. In dhc-1; mel-28 double mutants, proximal oocytes often fail to mature:**
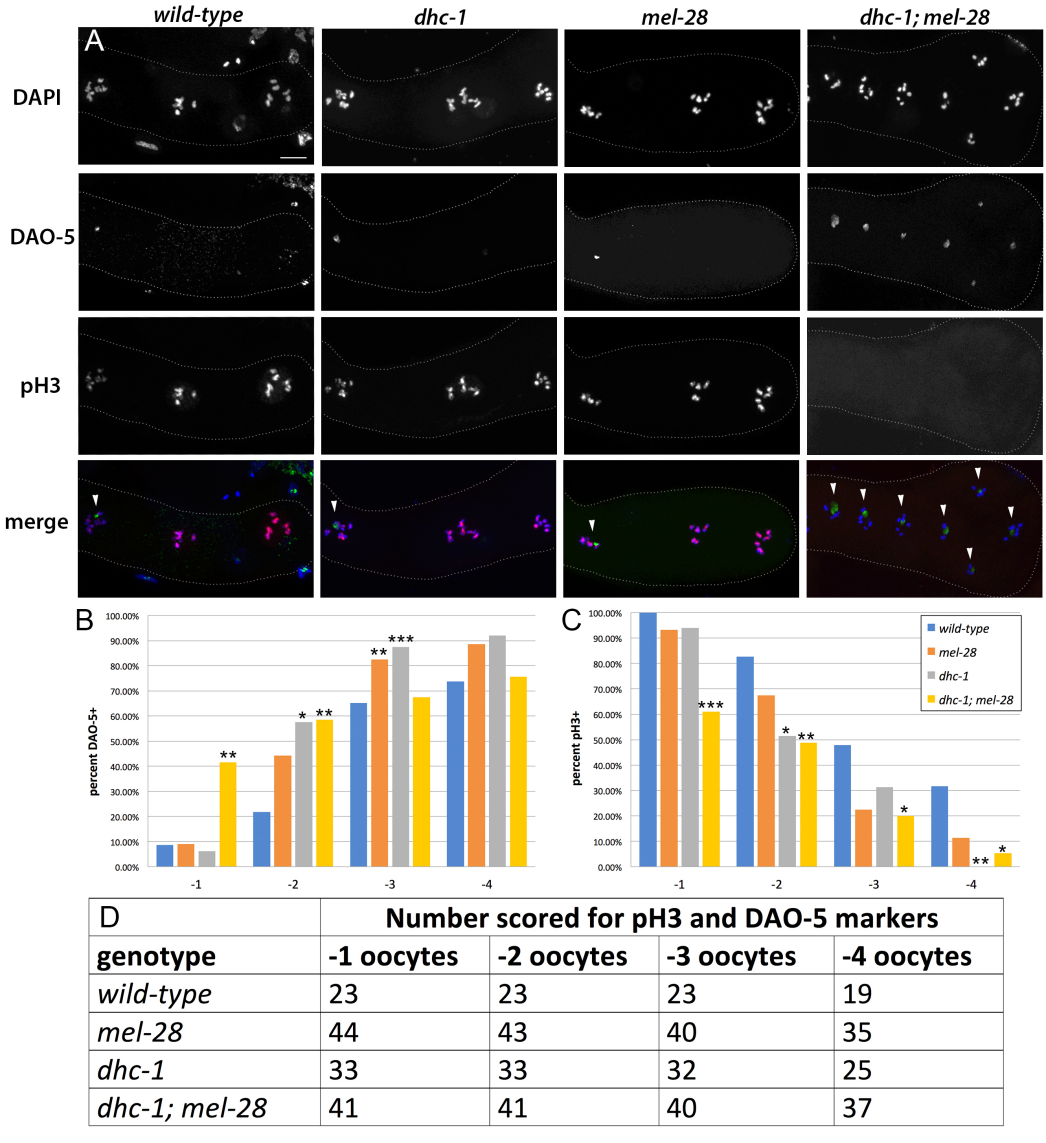
(A) Immunolocalizations of phosphorylated histone H3 and DAO-5 in proximal gonads captured using structured illumination. The dotted line outlines the proximal gonad, and the -1 oocyte is the right-most oocyte in all panels. In the merged images, blue indicates DAPI staining, red indicates phosphorylated histone H3, and green represents DAO-5. Arrowheads indicate DAO-5-positive nuclei in the merged images. Scale bar = 10 μM. (B) Frequency of DAO-5-positive nuclei in the four most proximal oocytes in each strain. (C) Frequency of nuclei with phosphorylated histone H3 in the four most proximal oocytes in each strain. We used a Fisher’s Exact test to compare each mutant with the wild-type at the equivalent oocyte position. *=p<0.05, ** =p<0.01, ***=p<0.001. (D) The number of oocytes scored at each position for each strain.

## Description

Most cellular processes require the coordinate activities of multiple gene products. These collaborations vary with different contexts. *mel-28* encodes a conserved multi-functional nucleoporin required for nuclear envelope integrity, the post-mitotic rebuilding of the nuclear pore, and the proper segregation of chromosomes during mitosis (Galy *et al.* 2006; Fernandez and Piano 2006). Loss of *mel-28* results in strict maternal-effect embryonic lethality. Thus homozygous mutant embryos produced from heterozygous mothers show typical wild-type development until they become adults and produce normal-sized broods of inviable embryos. This suggests that *mel-28* is required for embryonic development but is dispensable for post-embryonic development and adult processes such as fertility. To identify genes that function collaboratively with *mel-28*, we did an RNAi screen to find genes that cause fertility defects in *mel-28* mutant animals but not wild-type animals (Fernandez *et al.* 2014). Among the genes identified were those encoding components of the minus-end-directed microtubule motor dynein.

Cytoplasmic dynein is the major minus-end directed microtubule motor in eukaryotes. It consists of six subunits, each present within the complex as dimers (Pfister *et al.* 2006). The dynein heavy chain, encoded by *dhc-1* in *C. elegans*, is the largest subunit of the dynein complex. The head region of this subunit binds microtubules directly and is required for ATP hydrolysis. The tail domain binds to the remainder of the dynein subunits that in turn serve as adapters that permit interactions with cargoes. Dynein motor activity is required for diverse functions including transport of membrane-bound vesicles, centrosome separation, mitotic chromosome segregation, nuclear movement, virus transport, and organelle trafficking (Pfister *et al.* 2006).

To study the defects caused by simultaneous disruption of MEL-28 and dynein, we generated double mutants (Fernandez *et al.* 2014). We used a temperature-sensitive allele of *dhc-1(or283)* (Hamill *et al.* 2002; O’Rourke *et al.* 2007) in which dynein function is disabled at 26 °C and functions normally at 16 °C. *dhc-1; mel-28* double mutants shifted to the restrictive temperature as L4s produce a small brood size compared to each single mutant and compared to the wild-type (Fernandez *et al.* 2014).

In wild-type *C. elegans,* diakinesis-stage oocytes undergo meiotic maturation in response to maturation-promoting factor (MPF), which is in turn positively regulated by the phosphatase CDC-25.2 and negatively regulated by the Myt1 kinase WEE-1.3 (Burrows *et al.* 2006; Kim *et al.* 2010; Huelgas-Morales and Greenstein, 2018). Since defects in oocyte maturation cause fertility defects, we sought to determine whether the reduced brood size we observed in *dhc-1; mel-28* double mutants is coincident with oocyte maturity defects. To accomplish this, we used immunolocalizations to study oocyte maturity markers in *dhc-1; mel-28* mutants, *dhc-1* mutants, *mel-28* mutants, and wild-type animals.

In the proximal gonad of wild-type hermaphrodites, oocytes are aligned in a linear assembly-line fashion such that the -1 oocyte, which is the oocyte closest to the spermatheca, is the most mature and the next to be ovulated and fertilized (McCarter *et al.* 1999; Huelgas-Morales and Greenstein 2018). Oocytes more distal to the spermatheca are progressively less mature. As oocytes mature their nucleoli are dismantled and their chromatin accumulates phosphorylated histone H3 (Burrows *et al.* 2006). Thus, in wild-type animals, the -1 oocyte typically lacks the nucleolus and its chromatin has phosphorylated histone H3.

To determine whether the reduced fertility in *dhc-1; mel-28* double mutants is associated with oocyte maturity defects, we studied the proximal gonad using oocyte maturity markers. We used DAO-5 antibodies, which detect the Nopp140 component of the nucleolus (Korcekova *et al.* 2012; Hadwiger *et al.* 2010) as a marker for oocyte immaturity, and antibodies against phosphorylated histone H3 (Hendzel *et al.* 1997) as a marker for mature oocytes. In wild-type animals, *dhc-1* single mutants, and *mel-28* single mutants, the -1 oocyte rarely retained the nucleolar marker DAO-5 but nearly always had chromatin with phosphorylated histone H3. However, in *dhc-1; mel-28* double mutants, the -1 oocyte frequently retained the DAO-5 immaturity marker and failed to accumulate the phosphorylated histone H3 maturity marker (Figure 1A-D). This shows that in *dhc-1; mel-28* double mutants, oocytes occupying the most proximal positions do not always achieve mature status, suggesting that dynein and MEL-28 contribute in parallel to the maturity program in the oogenic gonad.

About 25% of the *dhc-1; mel-28* gonads had a disorganized proximal region with several oocytes clustered near the spermatheca instead of being aligned one-by-one (Figure 1A). In some cases two oocytes essentially occupied the same position; For example, in the *dhc-1; mel-28* gonad in Figure 1A, the -2 and -3 oocyte cannot be discerned. In all cases when the proximal gonads showed this disorganization, the oocytes occupying equivalent positions displayed the same combination of markers, making it irrelevant which oocyte was interpreted to be in which position. All gonads that showed this proximal clustering of oocytes also exhibited -1 oocyte maturity failure. In addition we observed some *dhc-1; mel-28* animals with immature proximal oocytes and no obvious morphological disorganization.

While reduced fertility in *dhc-1; mel-28* double mutants is a consistently penetrant phenotype (Fernandez *et al.* 2014), we observed the aberrant immature status of the -1 oocyte in only about 40% of the *dhc-1; mel-28* gonads we studied (Figure 1B and 1C). This suggests that oocyte maturity defects are not the primary cause of the low fertility phenotype. We favor the idea that the oocyte maturity defects described here are a secondary effect of a problem caused by the simultaneous compromise of *mel-28* and minus-end directed motor activity.

## Methods

L4 hermaphrodites were placed at 26°C overnight, then dissected on a polylysine-coated slide in Egg Buffer (25 mM HEPES pH 7.4, 118 mM NaCl, 48 mM KCl, 2 mM CaCl2, 2 mM MgCl2) with 2 mM levamisole. After freeze cracking, slides were placed in methanol at -20 °C for ten minutes, followed by acetone at -20 °C for five minutes, followed by an ice-cold acetone hydration series. After a PBS wash, slides were incubated with primary antibodies (1:200 Rabbit anti-phosphorylated histone H3 and 1:10 mouse anti-DAO-5 in PBS) in a humid chamber at room temperature overnight. Slides were washed three times in PBST and incubated with secondary antibodies (1:400) in PBS in a humid chamber for one hour. Slides were washed three times in PBST and mounted using Vectashield Plus Antifade Mounting medium with DAPI and sealed with nail polish. Imaging was done at 100X using a Keyence BZ-X800 microscope. We captured Z stacks using sectioning capture in each channel and generated a maximum intensity projection of the stack using Keyence analyzer software. Images were processed using Adobe Photoshop.

## Reagents

**Table:** 

***C. elegans* strains**		**availability**
AGF001	*mel-28(t1684)*/qC1 *dpy-19(e1259) glp-1(q339) qIs26* [*lag-2::GFP + rol-6(su1006)] III*	upon request
AGF035	*dhc-1(or283ts) I; mel-28(t1684)/*qC1 *dpy-19(e1259) glp-1(q339) [qIs26] III*	upon request
N2	wild type	CGC
EU1385	*dhc-1(or283ts)*	CGC

**antibodies**		**availability**
anti-DAO-5		Developmental Studies Hybridoma Bank https://dshb.biology.uiowa.edu/
anti-phosphorylated histone H3		Abcam
Goat anti-mouse FITC		Jackson
Goat anti-rabbit TRITC		Jackson
